# Clinical characteristics and salivary biomarkers of burning mouth syndrome

**DOI:** 10.1111/odi.14959

**Published:** 2024-04-15

**Authors:** Mi‐Sun Kong, Moon‐Jong Kim, Yoon‐Young Kim, Ji‐Youn Chang, Hong‐Seop Kho

**Affiliations:** ^1^ Department of Oral Medicine and Oral Diagnosis School of Dentistry and Dental Research Institute Seoul National University Seoul South Korea; ^2^ Department of Oral Medicine Gwanak Seoul National University Dental Hospital Seoul South Korea; ^3^ Institute on Aging Seoul National University Seoul South Korea

**Keywords:** biomarker, burning mouth syndrome, saliva

## Abstract

**Objectives:**

To investigate the clinical characteristics and salivary biomarkers in each type of burning mouth syndrome (BMS) patients.

**Materials and Methods:**

Ninety‐eight postmenopausal female patients with BMS were included. Fifty and 21 patients were assigned to the primary and secondary groups, respectively. Twenty‐seven patients with both primary and secondary characteristics were assigned to the intermediate group. Comprehensive clinical characteristics and salivary biomarkers were analyzed.

**Results:**

Significant differences in age, proportion of hyposalivator patients based on unstimulated whole saliva (UWS), symptom distribution, severties of burning sensation and effect of oral complaints in daily life (Eff‐life), and positive symptom distress index (PSDI) were observed among the three groups. The primary group had significant higher UWS flow rate, fewer UWS hyposalivator proportions, and lesser severity of Eff‐life than the secondary group. The intermediate group had significantly greater intensities of burning sensation and Eff‐life and higher PSDI score than did the primary group. The primary group had significantly higher cortisol and dehydroepiandrosterone (DHEA) levels in stimulated whole saliva than did the secondary group.

**Conclusions:**

This study's findings show that clinical characteristics differentiate each BMS type. Cortisol and DHEA levels are potential salivary biomarkers for discriminating between the primary and secondary types of BMS.

## 1 INTRODUCTION

Burning mouth syndrome (BMS) is a chronic orofacial pain condition characterized by intraoral burning pain and/or dysesthesia. A comprehensive evaluation is necessary to exclude possible local and systemic factors responsible for BMS symptoms. If any local and/or systemic factors are involved, it is considered burning mouth symptoms not true BMS, which has been called as the secondary BMS. True primary BMS without any related local and/or systemic factors is supposedly peripheral and/or central neuropathy (Klasser et al., 2016; Scala et al., 2003).

Previous studies have mostly focused on the clinical characteristics and pathophysiology of the primary BMS patients selected by heterogeneous criteria compared with controls (Dias Fernandes et al., 2009; de Souza et al., 2015; Jääskeläinen, 2018; Kim et al., 2012; Lauria et al., 2005; Nosratzehi et al., 2017; Suh et al., 2009). The lack of consensus on definite inclusion/exclusion criteria for the primary BMS (Ariyawardana et al., 2019) has resulted in limited information comparing the characteristics of the primary and secondary BMS (Eliav et al., 2007; Khawaja et al., 2020; Kishore et al., 2022; Terai & Shimahara, 2007). Furthermore, patients presenting with both the primary and secondary BMS characteristics were less considered (Hato et al., 2021) because of exclusion or mis‐inclusion in the secondary BMS (Khawaja et al., 2020; Kishore et al., 2022).

Studies using saliva as a diagnostic modality have also been applied to BMS. Based on BMS pathophysiology, previous studies have investigated salivary profiles such as pain‐ or stress‐related (Amenábar et al., 2008; Dias Fernandes et al., 2009; Kim et al., 2012; Nosratzehi et al., 2017), hormonal (Kim et al., 2012; Woda et al., 2009), inflammatory, and oxidative stress (Suh et al., 2009) biomarkers.

The inclusion/exclusion criteria, standardization of collection, processing, and analytic procedures for saliva samples (Nam et al., 2019; Tighe et al., 2015), and inclusion of valid analytic results (e.g. level of blood contamination in saliva samples) (Kang & Kho, 2019; Suh et al., 2009) could affect the results of salivary biomarker studies targeting patients with BMS. Because the concentrations of most analytes are much higher in blood than in saliva (Nunes et al., 2015), blood contamination of saliva samples can cause significant errors in analytical results (Kang et al., 2018; Kang & Kho, 2019; Suh et al., 2009), but this was not considered in most studies. This is especially important in patients with BMS who are usually middle‐aged and elderly with periodontal disease (Kang et al., 2018; Kang & Kho, 2019).

Therefore, in this study, to overcome the problems in previous studies, patients with BMS were classified into three groups [primary, secondary, and both primary and secondary (intermediate)] using strict inclusion/exclusion criteria and attempted to include only valid results of salivary biomarkers considering blood contamination of saliva samples. This study aimed to (1) compare clinical characteristics and salivary biomarkers between patients with the primary and secondary BMS, (2) compare patients having both the primary and secondary characteristics with patients with the primary or secondary BMS, and (3) investigate the relationship between clinical characteristics and salivary biomarkers.

## 2 MATERIALS AND METHODS

This prospective study was conducted at the Department of Oral Medicine, Seoul National University Dental Hospital from November 1, 2017 to October 31, 2020. One hundred consecutive patients with oral burning pain or dysesthesia participated in this study. The research protocol was approved by the IRB of Seoul National University Dental Hospital (CRI17008 & CRI19016). Every patient was informed about the study and signed written consents.

### 2.1 Inclusion and exclusion criteria

The inclusion criteria included a complaint of oral burning or dysesthetic sensation and post‐menopause female patients. The exclusion criteria included premenopause female patients, male patients, and patients with difficulty answering the questionnaire. To minimize the influence of confounding factors, only typical BMS patients of post‐menopausal females were included.

### 2.2 Clinical evaluation procedures and saliva collection

All participants underwent standardized diagnostic examinations, including oral examination, panoramic radiography, and psychological evaluation using the Symptom Checklist‐90‐Revised (SCL‐90‐R), Candida culture test, laboratory blood tests, measurement of salivary flow rates [unstimulated (UWS) and stimulated whole saliva (SWS)], BMS questionnaire, and interview. A detailed description of the diagnostic procedures has been previously described (Kim et al., 2018, 2020). Briefly, the BMS questionnaire included questions on sociodemographic characteristics, clinical characteristics including duration, distribution, area, and diurnal pattern of oral symptoms, modulating factors (aggravating and relieving factors), and insomnia. The intensity of each type of symptom was assessed using a visual analog scale (VAS) (0–10, with 10 being the most extreme symptom imaginable). The subjective assessment of sleep quality was evaluated using a VAS (0–10, with 10 being patients who could not sleep at all). To examine the individual severity of insomnia in detail, the insomnia severity index (ISI) was also used (Bastien et al., 2001; Cho et al., 2014).

To evaluate the presence of oral candidiasis, the “Candida detector (Kamemizu Chemical Industry Co., Osaka, Japan)” was used. Blood tests were conducted to exclude possible systemic factors related to BMS symptoms and detailed items have been described in a previous study (Kim et al., 2018).

Saliva was collected between 9:00 and 11:00 a.m. Participants were instructed to avoid eating or drinking and performing oral hygiene activities for 2 h before sample collection. UWS was collected using the spitting method for 10 min. SWS was collected for 5 min through the chewing of paraffin wax. UWS and SWS samples were centrifuged at 4000×*g* for 20 min at 4°C. The resulting supernatants were aliquoted and stored at −70°C until analysis.

### 2.3 Classification of patients

**FIGURE 1:**
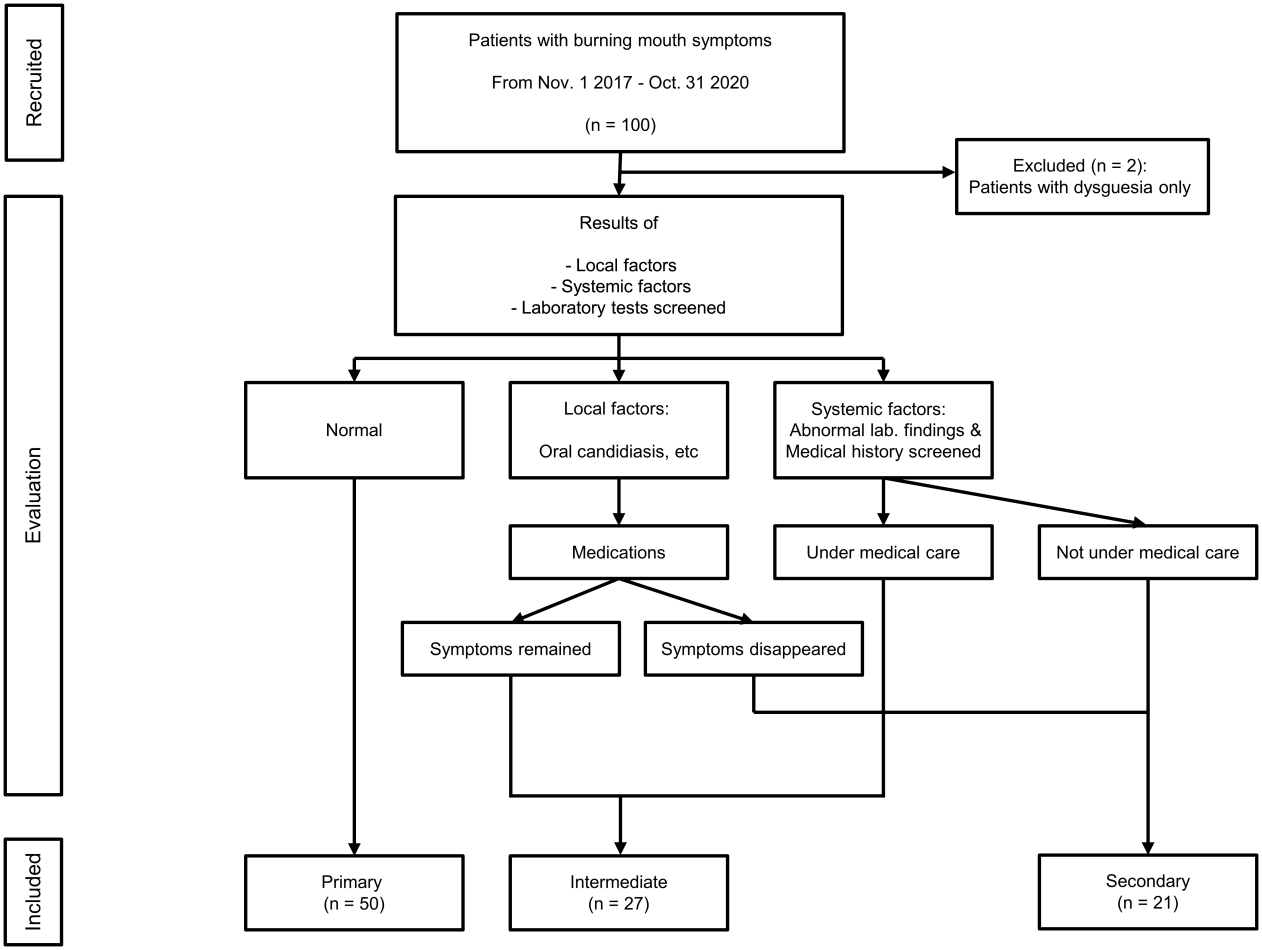
Flow diagram of the classification of patients with burning mouth symptoms.

Figure 1 shows a flow diagram of the evaluation and classification of patients. After thoroughly reviewing medical records and the results of the diagnostic examinations of the 100 patients, two patients with dysgeusia symptoms only were excluded. Then, 98 patients were finally eligible for the study and classified into three groups: the “primary,” “secondary,” and “intermediate” groups; the intermediate group included patients with both the primary and secondary characteristics. Patients with BMS symptoms following treatment with local factors and/or those receiving medical care for systemic factors were included in the intermediate group.

Patients without any local and/or systemic factors were classified into the primary group (*n* = 50). The remaining patients were classified into the intermediate or secondary groups based on laboratory findings, medical history, and treatment response to the causative factors. Twenty‐one patients responding well to the treatment of local factors and/or not receiving medical care for systemic factors were classified as the secondary group. The remaining 27 patients were classified as the intermediate group (Figure 1 and Table S1).

### 2.4 Analyses of salivary biomarkers

Total protein, inflammatory and oxidative stress biomarkers, and hormones were analyzed in the UWS and SWS samples. The total protein concentration was measured using the Pierce BCA assay (Thermo Fisher Scientific, Rockford, IL). C‐reactive protein (CRP), IL‐1β, and IL‐6 were analyzed as inflammatory biomarkers, and 8‐hydroxy‐2′‐deoxyguanosine (8‐OHdG), malondialdehyde (MDA), and total antioxidant capacity (TAC) were analyzed as oxidative stress biomarkers. The concentrations of inflammatory and oxidative stress biomarkers were determined using an immunoassay (CRP, IL‐1β, and IL‐6, Salimetrics, State College, PA; 8‐OHdG, Abcam, Cambridge, MA; MDA, Cell Biolabs, San Diego, CA) or an assay for measuring copper ion reduction activity (TAC, Abcam). Cortisol, dehydroepiandrosterone (DHEA), 17β‐estradiol, and progesterone concentrations were measured using immunoassay (Salimetrics). The cortisol/DHEA ratio was also calculated. Transferrin concentration in saliva samples was measured to assess the degree of blood contamination (Salimetrics). If the transferrin concentration was 2 mg/dL or higher, it was considered highly contaminated by blood. All assays were performed in duplicate.

### 2.5 Statistical analysis

The Kolmogorov–Smirnov test was used to determine data normality. To analyze differences among the three groups, ANOVA, or the Kruskal–Wallis test were used for continuous variables. Pearson's chi‐squared or Fisher's exact tests were used for categorical variables.

Because this study primarily aimed to compare between the primary and secondary groups, analyses to compare variables between these groups were performed even in cases of no significance among the three groups. The results of the intermediate group were compared with those of the primary and secondary groups. To analyze differences between the two groups, the Student's *t*‐test or Mann ‐Whitney *U*‐tests were used for continuous variables and the Pearson's chi‐squared or Fisher's exact tests were used for categorical variables. Spearman's correlation analysis with Bonferroni correction was used to analyze the relationships between variables. A *p* value less than 0.05 was considered statistically significant.

Principal component analysis (PCA) was used to assess similarities among the three groups. To perform PCA, variables with significant differences between the primary and secondary groups were selected and used. PCA results were visualized by representing a biplot with variable vectors to illustrate each variable's contribution and direction to the principal components. PCA was implemented using the PCA class from the scikit‐learn library in Python.

## 3 RESULTS

### 3.1 Clinical and sociodemographic characteristics

Table 1 shows the patients' clinical characteristics. There was a statistically significant difference in age across the three groups (*p* = 0.011). The patients in the intermediate group were significantly older than those in the primary group (*p* = 0.002). The flow rate of UWS in the secondary group was significantly higher than that in the primary group (*p* = 0.028). When the objective hyposalivation criteria, UWS ≤0.1 or SWS ≤0.7 mL/min, were considered, the percentages of hyposalivators in the secondary and intermediate groups were significantly higher than those in the primary group (*p* = 0.004 and 0.024, respectively). No significant difference was observed in BMI or level of education (Table S2).

**TABLE 1:** Clinical characteristics of patients with burning mouth symptoms; mean ± SD, median [IQR], *n* (%).

	Primary (*n* = 50)	Intermediate (*n* = 27)	Secondary (*n* = 21)	*p* Value	*p* Value		
					P vs. S	P vs. I	S vs. I
Age (years)	63.3 ± 6.1	68.3 ± 7.6	65.8 ± 7.9	0.011*	0.153	0.002*	0.267
Flow rate (mL/min)							
UWS	0.17 [0.14]	0.16 [0.23]	0.11 [0.12]	0.066	0.028*	0.405	0.429
SWS	0.83 [0.47]	0.81 [0.85]	0.82 [0.81]^a^	0.957	0.765	0.439	0.533
UWS ≤0.1 mL/min	6 (12.0)	9 (33.3)	10 (47.6)	0.004*	0.004*	0.024*	0.315
SWS ≤0.7 mL/min	14 (28.0)	12 (44.4)	8 (40.0)^a^	0.308	0.329	0.145	0.761
Duration (months)	12.0 [20.0]	24.0 [56.0]	6.0 [24.5]	0.298	0.557	0.168	0.227
Range	2–180	2–300	2–120				
Distribution							
Unilateral	5 (10.0)	10 (37.0)	6 (28.6)	0.015*	0.072	0.004*	0.357
Bilateral	45 (90.0)	17 (63.0)	15 (71.4)				
Area							
Tongue only	33 (66.0)	11 (40.7)	13 (61.9)	0.095	0.902	0.094	0.334
Tongue with other areas	15 (30.0)	15 (55.6)	7 (33.3)				
Other areas except the tongue	2 (4.0)	1 (3.7)	1 (4.8)				
Diurnal pattern							
Increasing	19 (38.0)	10 (37.0)	7 (33.3)	0.169	0.061	1	0.099
Decreasing	1 (2.0)	0 (0.0)	3 (14.3)				
Continuous	15 (30.0)	9 (33.3)	9 (42.9)				
Irregular	15 (30.0)	8 (29.6)	2 (9.5)				

*Note*: The ANOVA or Kruskal–Wallis test was used for continuous variables to analyze the differences across the three groups, and the Student's *t*‐test or Mann–Whitney *U*‐test was used for continuous variables between the groups. Pearson's chi‐squared test or Fisher's exact test was used to analyze differences between categorical variables.

Abbreviations: I, intermediate type of burning mouth syndrome (BMS); IQR, interquartile range; P, primary type of BMS; S, secondary type of BMS; SD, standard deviation; SWS, stimulated whole saliva; UWS, unstimulated whole saliva.

^a^ SWS could not be collected from one patient who could not chew the paraffin wax because of missing posterior teeth.

* *p* < 0.05.

### 3.2 Duration, distribution, area, and diurnal pattern of oral symptoms

Table 1 shows the duration, distribution, area, and diurnal pattern of oral symptoms. No significant differences were found in the duration of oral symptoms among the three groups (*p* = 0.298). Most patients in the three groups reported a typical bilateral distribution of oral symptoms, with the highest percentage (90.0%) in the primary group. The ratio of unilateral to bilateral distribution differed significantly among the three groups (*p* = 0.015), particularly between the primary and intermediate groups (*p* = 0.004).

More than half of the patients in the primary and secondary groups reported oral symptoms on the tongue only (primary, 66.0%; secondary, 61.9%), while more than half of the patients in the intermediate group reported oral symptoms on the tongue and other oral mucosal areas (55.6%). Regarding the diurnal pattern of oral symptoms, a decreasing pattern was very rare in the primary and intermediate groups, whereas an irregular pattern was rare in the secondary group.

### 3.3 Prevalence and intensity of the oral symptoms

Table 2 shows the prevalence and intensity of oral symptoms. The prevalence of burning, aching, stinging, and numbness symptoms did not differ across the three groups. The prevalence of taste disturbance, xerostomia, sore throat, the effect of oral complaints on daily life (Eff‐life), and symptom triad (burning sensation, xerostomia, and taste disturbance) did not differ, either.

**TABLE 2:** Prevalence and intensity of oral symptoms in patients with burning mouth symptoms; median [IQR], *n* (%).

	Primary (*n* = 50)	Intermediate (*n* = 27)	Secondary (*n* = 21)	*p* Value	*p* Value		
					P vs. S	P vs. I	S vs. I
Prevalence							
Burning	43 (86.0)	26 (96.3)	19 (90.5)	0.409	0.716	0.248	0.574
Aching	24 (48.0)	13 (27.1)	11 (52.4)	0.940	0.736	0.990	0.771
Stinging	15 (30.0)	8 (29.6)	4 (19.0)	0.616	0.341	0.973	0.401
Numbness	8 (16.0)	4 (14.8)	5 (23.8)	0.728	0.507	1	0.477
Taste disturbance	24 (48.0)	16 (59.3)	13 (61.9)	0.460	0.284	0.345	0.853
Xerostomia	40 (80.0)	20 (74.1)	16 (76.2)	0.826	0.756	0.550	0.867
Sore throat	20 (40.0)	11 (40.7)	5 (23.8)	0.382	0.192	0.950	0.217
Eff‐life	39 (79.6)	24 (88.9)	20 (95.2)	0.219	0.154	0.359	0.621
Symptom triad	18 (36.0)	14 (51.9)	10 (47.6)	0.359	0.361	0.178	0.771
Intensity (VAS)							
Burning	6.0 [4.0]	8.0 [2.0]	8.0 [3.5]	0.004*	0.075	0.001*	0.302
Aching	0 [6.0]	0 [7.0]	4.0 [7.5]	0.637	0.335	0.606	0.816
Stinging	0 [3.5]	0 [6.0]	0 [0]	0.493	0.328	0.686	0.253
Numbness	0 [0]	0 [0]	0 [1.0]	0.606	0.416	0.806	0.352
Taste disturbance	0 [5.0]	5.0 [8.0]	5.0 [7.0]	0.157	0.187	0.079	0.732
Xerostomia	5.0 [5.5]	6.0 [8.0]	5.0 [6.0]	0.624	0.580	0.567	0.333
Sore throat	0 [5.0]	0 [4.0]	0 [4.0]	0.622	0.394	0.504	0.697
Eff‐life	6.0 [6.0]	8.0 [3.0]	8.0 [3.0]	0.028*	0.048*	0.021*	0.599

*Note*: Symptom triad means three common symptoms found in patients with BMS, including burning sensation, xerostomia, and taste disturbance. The Kruskal–Wallis test was used for continuous variables to analyze differences across the three groups, and the Mann–Whitney *U*‐test was used for continuous variables between the groups. Pearson's chi‐squared test or Fisher's exact test was used to analyze differences in categorical variables.

Abbreviations: Eff‐life, effect of oral complaints on daily life; I, intermediate type of burning mouth syndrome (BMS); IQR, interquartile range; P, primary type of BMS; S, secondary type of BMS; VAS, visual analog scale.

* *p* < 0.05.

Regarding the severity of oral symptoms, burning and Eff‐life differed significantly among the three groups (*p* = 0.004 and 0.028, respectively). The VAS score for burning sensation in the intermediate group was significantly higher than that in the primary group (*p* = 0.001). The VAS scores of Eff‐life in the intermediate and secondary groups were significantly higher than those in the primary group (*p* = 0.021 and 0.048, respectively).

### 3.4 Initiating, aggravating, and relieving factors of oral symptoms

Modulating factors of oral symptoms were analyzed (Tables S3—1–3). The secondary group reported “systemic disease” as an initiating factor more frequently than did the intermediate group (*p* = 0.040). Regarding aggravating factors, the “feeling of anger” proportion differed significantly among the groups (*p* = 0.009), with a significantly higher proportion in the intermediate than in the other groups. “Toothpaste” differed significantly between the primary and secondary groups (*p* = 0.047), and “salty food” differed significantly between the primary and intermediate groups (*p* = 0.019). Regarding relieving factors, “cold food” differed significantly between the primary and secondary groups (*p* = 0.035).

### 3.5 Symptom checklist‐90‐revised (SCL‐90‐R)

The median t‐scores of the nine symptom dimensions and three global indices in the SCL‐90‐R were within the normal range (under 60) in the three groups. The positive symptom distress index (PSDI) was the only item that differed significantly among the groups (*p* = 0.008), with a significantly higher score in the intermediate group than in the primary group (*p* = 0.002) (Table S4).

### 3.6 Sleep questionnaire results

The VAS score for quality of sleep and all items in the ISI questionnaire did not differ significantly among the groups. The percentage of patients with clinically moderate to severe insomnia was 26.0%–33.4% (Table S5).

### 3.7 Characteristics of salivary biomarkers

Tables 3 and 4 show the results for salivary biomarkers in UWS and SWS samples, respectively. No significant differences were found among the three groups for all examined salivary biomarkers in both types of saliva samples. No differences were found between any two groups in the UWS samples. However, in the SWS samples, the cortisol and DHEA concentrations were significantly higher in the primary group than in the secondary group (cortisol, *p* = 0.036; DHEA, *p* = 0.039). However, these differences in cortisol and DHEA levels were not found between the primary and intermediate groups, as well as between the intermediate and secondary groups.

**TABLE 3:** Characteristics of salivary biomarkers in unstimulated whole saliva samples from patients with burning mouth symptoms; median [IQR].

	Primary (*n* = 33)^a^		Intermediate (*n* = 13)^a^		Secondary (*n* = 10)^a^		*p* Value	*p* Value		
	Median [IQR]	*n*	Median [IQR]	*n*	Median [IQR]	*n*		P vs. S	P vs. I	S vs. I
Flow rate (mL/min)	0.13 [0.12]	33	0.20 [0.14]	13	0.14 [0.35]	10	0.160	0.279	0.074	0.574
Transferrin (mg/dL)	0.74 [0.85]	33	0.42 [0.79]	13	0.61 [0.73]	10	0.171	0.336	0.079	0.402
Total protein (mg/mL)	1.65 [0.62]	33	1.69 [0.54]	13	1.76 [0.73]	10	0.930	0.931	0.742	0.710
Inflammatory marker										
CRP (pg/mL)	919.7 [1446.9]	33	723.1 [911.2]	13	305.3 [725.7]	10	0.441	0.262	0.951	0.203
IL‐1β (pg/mL)	480.3 [792.2]	33	285.1 [936.5]	13	474.1 [758.9]	10	0.510	0.388	0.335	0.852
IL‐6 (pg/mL)	3.03 [5.63]	33	3.69 [3.14]	13	7.42 [12.15]	10	0.457	0.239	0.779	0.306
Oxidative stress marker										
8‐OHdG (ng/mL)	18.7 [14.0]	33	11.8 [26.3]	13	12.8 [12.8]	10	0.315	0.128	0.807	0.292
MDA (pmol/mL)	130.7 [309.2]	33	90.0 [402.1]	13	69.8 [256.1]	10	0.461	0.206	0.634	0.577
TAC (mmol/L)	2.46 [0.80]	33	2.49 [0.84]	13	2.31 [1.04]	10	0.991	0.931	0.990	0.852
Hormones										
Cortisol (μg/dL)	0.15 [0.11]	32	0.17 [0.19]	13	0.15 [0.13]	10	0.940	0.712	0.841	0.877
DHEA (pg/mL)	31.4 [34.7]	32	21.7 [67.6]	13	27.6 [36.5]	9	0.882	0.637	0.745	0.973
Cortisol/DHEA ratio	51.3 [68.9]	32	39.1 [195.6]	13	64.7 [43.0]	9	0.803	0.529	1.000	0.570
Progesterone (pg/mL)	25.9 [44.9]	29	13.8 [47.6]	13	22.0 [21.1]	9	0.502	0.525	0.283	0.570
17β‐Estradiol (pg/mL)	1.09 [0.46]	31	1.05 [0.53]	13	0.96 [1.02]	9	0.546	0.323	0.898	0.301

*Note*: The analysis results were not obtained for some biomarkers because of a shortage of saliva samples. The Kruskal–Wallis test was used for continuous variables to analyze the differences across the three groups, and the Mann–Whitney *U*‐test was used for continuous variables between the groups.

Abbreviations: CRP, C‐reactive protein; DHEA, dehydroepiandrosterone; I, intermediate type of burning mouth syndrome (BMS); IL, interleukin; IQR, interquartile range; MDA, malondialdehyde; 8‐OHdG, 8‐hydroxy‐2′‐deoxyguanosine; P, primary type of BMS; S, secondary type of BMS; TAC, total antioxidant capacity.

^a^ Unstimulated whole saliva samples were not collected in three patients in the primary group, five in the intermediate group, and four in the secondary group because of severe oral dryness (flow rate, undetectable, 0 mL/min). Fourteen saliva samples in the primary group, nine in the intermediate group, and seven in the secondary group were excluded from the analyses because of the high levels of blood contamination (transferrin ≥2.0 mg/dL) in the saliva samples.

**TABLE 4:** Characteristics of salivary biomarkers in stimulated whole saliva samples from patients with burning mouth symptoms; median [IQR].

	Primary (*n* = 48)^a^		Intermediate (*n* = 24)^a^		Secondary (*n* = 20)^a^		*p* Value	*p* Value		
	Median [IQR]	*n*	Median [IQR]	*n*	Median [IQR]	*n*		P vs. S	P vs. I	S vs. I
Flow rate (mL/min)	0.71 [0.55]	48	0.86 [0.76]	24	0.71 [0.89]	20	0.955	0.957	0.816	0.750
Transferrin (mg/dL)	0.53 [0.50]	48	0.49 [0.74]	24	0.38 [0.61]	20	0.762	0.623	0.725	0.458
Total protein (mg/mL)	1.21 [0.48]	48	1.43 [0.72]	24	1.26 [0.72]	20	0.234	0.941	0.085	0.268
Inflammatory marker										
CRP (pg/mL)	323.7 [1392.2]	48	473.1 [915.8]	24	561.5 [1488.7]	20	0.930	0.706	0.957	0.777
IL‐1β (pg/mL)	360.7 [396.7]	48	188.6 [325.0]	22	277.8 [316.5]	19	0.575	0.381	0.418	0.896
IL‐6 (pg/mL)	2.77 [2.58]	48	3.82 [3.98]	22	3.07 [2.64]	18	0.298	0.604	0.134	0.328
Oxidative stress marker										
8‐OHdG (ng/mL)	10.9 [6.6]	48	11.7 [11.7]	22	11.5 [10.8]	18	0.959	0.796	0.960	0.786
MDA (pmol/mL)	55.1 [185.4]	48	52.7 [184.9]	22	67.3 [379.7]	18	0.905	0.840	0.800	0.605
TAC (mmol/L)	1.97 [0.81]	48	2.18 [0.99]	22	1.78 [1.04]	18	0.155	0.555	0.092	0.103
Hormones										
Cortisol (μg/dL)	0.14 [0.10]	48	0.16 [0.17]	21	0.10 [0.11]	18	0.105	0.036*	0.860	0.108
DHEA (pg/mL)	44.8 [51.5]	47	44.1 [69.9]	24	21.3 [23.3]	20	0.094	0.039*	0.770	0.077
Cortisol/DHEA ratio	34.1 [68.9]	47	38.5 [77.9]	21	46.7 [64.8]	18	0.806	0.860	0.503	0.735
Progesterone (pg/mL)	24.3 [30.0]	47	18.2 [40.0]	24	15.4 [25.4]	20	0.546	0.273	0.593	0.654
17β‐Estradiol (pg/mL)	1.02 [0.86]	47	1.16 [1.03]	24	0.92 [0.94]	20	0.164	0.551	0.155	0.056

*Note*: The analysis results were not obtained for some biomarkers because of a shortage of saliva samples. The Kruskal–Wallis test was used for continuous variables to analyze differences across the three groups, and the Mann–Whitney *U*‐test was used for continuous variables between the groups.

Abbreviations: CRP, C‐reactive protein; DHEA, dehydroepiandrosterone; I, intermediate type of burning mouth syndrome (BMS); IL, interleukin; IQR, interquartile range; MDA, malondialdehyde; 8‐OHdG, 8‐hydroxy‐2′‐deoxyguanosine; P, primary type of BMS; S, secondary type of BMS; TAC, total antioxidant capacity.

^a^ Stimulated whole saliva samples were not collected in one patient in the secondary group who could not chew the paraffin wax because of missing posterior teeth. Two saliva samples in the primary group and three in the intermediate group were excluded from the analyses because of the high levels of blood contamination (transferrin ≥2.0 mg/dL) in the saliva samples.

* *p* < 0.05.

### 3.8 Correlations between clinical characteristics and salivary biomarkers

The intensities of oral symptoms were not correlated with the levels of any biomarkers in the UWS samples (Table S6—1). No correlations were found between the intensities of almost all oral symptoms and biomarker levels in the SWS samples, either. Only the intensity of the aching symptom correlated significantly with the cortisol level in the SWS samples (*r*_s_ = −0.426) (Table S6–2). All items of SCL‐90‐R were not significantly correlated with the biomarker levels in the UWS and SWS samples (Tables S7—1–2).

### 3.9 Principal component analysis

**FIGURE 2:**
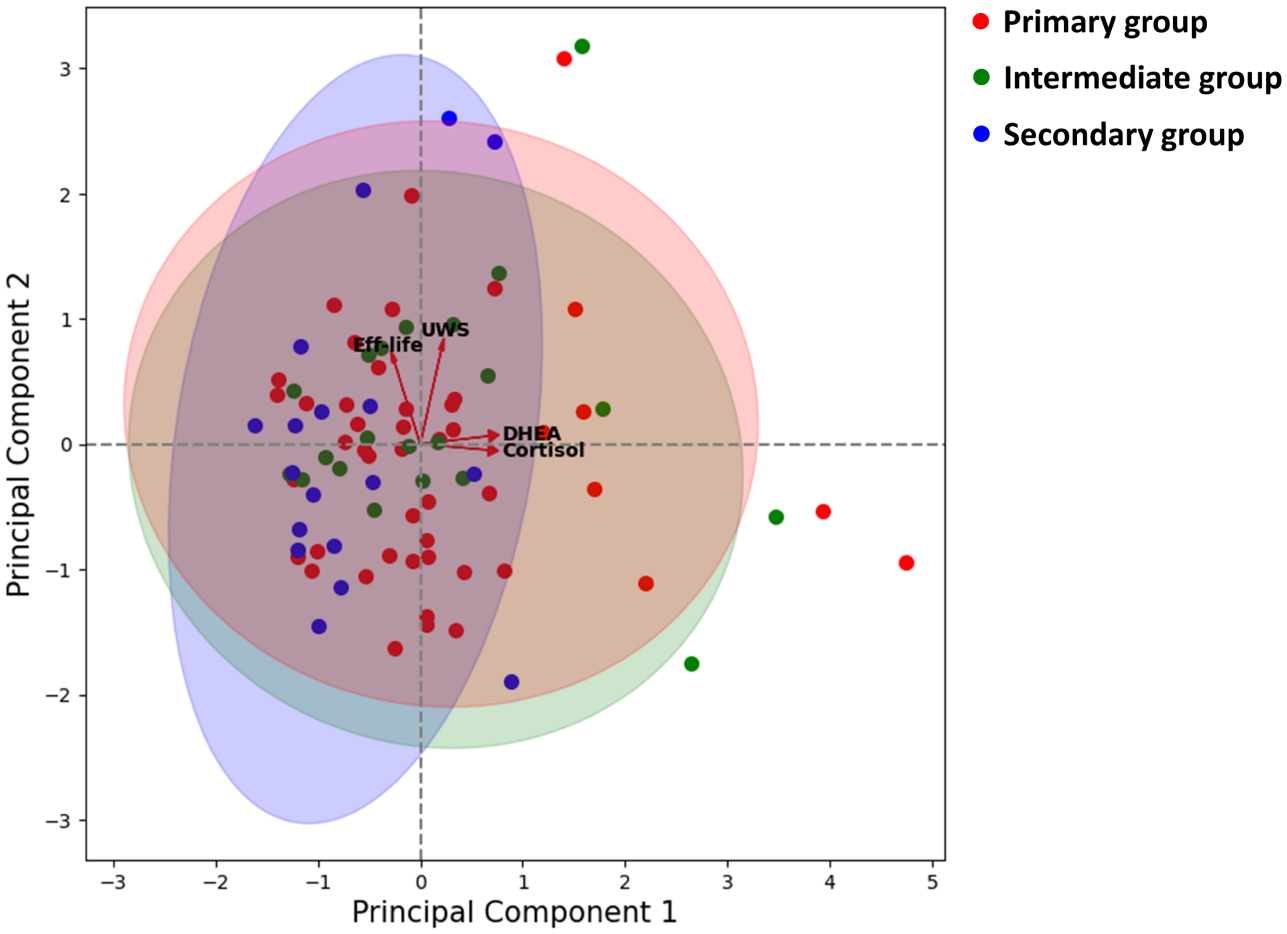
Biplot of the results of the principal component analysis. DHEA, dehydroepiandrosterone; Eff‐life, effect of oral complaints on daily life; UWS, flow rate of unstimulated whole saliva.

PCA was conducted using four variables (the flow rate of UWS, intensity of Eff‐life, and cortisol and DHEA levels in SWS) which were significant in the comparison between the primary and secondary groups. The eigenvalues of principal components (PC) 1 and 2 were 34.8% and 25.5%, respectively. In the biplot, the three groups were not clearly discriminable and largely overlapped. The distributions of the primary and intermediate groups were similar but differed from those of the secondary group. These differences were noticeable in the direction of cortisol and DHEA concentration vectors (Figure 2).

## 4 DISCUSSION

To the best of our knowledge, this is the first study to differentiate clinical characteristics and salivary biomarkers of various BMS types (primary, secondary, and intermediate) classified based on strict criteria and considering blood contamination of saliva samples.

The results of the present study showed important clinical characteristics that distinguish the primary BMS from the secondary BMS. Patients with the primary BMS have many characteristics of typical BMS, such as burning type of pain on the tongue as the main symptom, bilateral distribution of symptoms, and normosalivators with xerostomic symptoms (Kim et al., 2020; Suh et al., 2009). The low‐flow rate of UWS in the secondary group could be explained by accompanying diseases and medications (Sreebny & Vissink, 2010).

Among salivary biomarkers, cortisol and DHEA levels in SWS were higher in the primary BMS than in the secondary. Increased cortisol levels may reflect how psychological stress including anxiety and depression, are frequently associated with chronic pain such as BMS (Amenábar et al., 2008; Koike et al., 2014). DHEA is reportedly involved in the regulation of sensory and neuropathic pain by activating neuronal excitability and could increase pain sensitivity (Dias Fernandes et al., 2009). However, inconsistent results regarding salivary cortisol (Amenábar et al., 2008; de Souza et al., 2015; Kim et al., 2012; Nosratzehi et al., 2017) and DHEA levels (Dias Fernandes et al., 2009; Kim et al., 2012) in patients with BMS have been found across previous studies. Some studies reported an increase in salivary cortisol level in patients with BMS (Amenábar et al., 2008; Kim et al., 2012), while others reported no difference (de Souza et al., 2015; Nosratzehi et al., 2017). There was a study reporting a decrease in salivary DHEA level in patients with BMS (Dias Fernandes et al., 2009), while another study found no difference (Kim et al., 2012). These inconsistent results could be due to the different inclusion criteria of the primary BMS, inclusion of male patients (Amenábar et al., 2008; de Souza et al., 2015; Nosratzehi et al., 2017), and the lack of consideration for blood contamination in saliva samples (Amenábar et al., 2008; de Souza et al., 2015; Dias Fernandes et al., 2009; Nosratzehi et al., 2017). In addition, all these previous studies compared between patients with BMS and controls without BMS, so there were limitations in direct comparisons with our present study.

In this study, we investigated the characteristics of the intermediate group which was classified as exclusion criteria in most previous studies investigating patients with the primary BMS compared with controls (de Souza et al., 2015; Dias Fernandes et al., 2009; Kim et al., 2012; Lauria et al., 2005; Nosratzehi et al., 2017; Suh et al., 2009) or misclassified as the secondary group in other studies (Khawaja et al., 2020; Kishore et al., 2022). In terms of the clinical characteristics, the intermediate group differed significantly from the primary group, whereas for salivary profiles, the intermediate group differed significantly from the secondary group. These could imply that the intermediate group is more related to the primary group pathophysiologically but underlying local and/or systemic factors might have influenced the expressed clinical pattern of oral burning sensations. In the biplot of PCA analysis, the intermediate group was distributed more similarly to the primary group than to the secondary group, and this difference was related to the levels of cortisol and DHEA. The severities of the burning sensation and Eff‐life were highest in the intermediate group. This may explain the highest PSDI score in the intermediate group in which patients' perception of distress and tendency to maximize the pain caused by BMS was high, and the severity of pain and/or discomfort caused by BMS significantly affected their quality of life with increased psychological features. Although not significant, the intermediate group had the longest duration of BMS symptoms, explaining the higher chance of association with psychological disturbances (Trombelli et al., 1994). These results show that the intermediate group had characteristics of the primary and secondary BMS with higher levels of pain and a lower quality of life, as well as increased psychological features. Therefore, in the future studies, considering the intermediate group may help narrow down the true BMS by more accurately expressing the characteristics of patients with the primary BMS compared to patients with burning mouth symptoms.

In the correlation analyses, the intensity of aching symptom correlated negatively with the cortisol level in SWS. This finding suggests that stress affects the subjective awareness of their discomforts in patients with BMS. Persistent pain and hypocortisolism may occur because of an extended stress response (Hannibal & Bishop, 2014), suggesting that the types and duration of symptoms need to be considered in psychoendocrinological interactions. These findings could explain the inconsistent results regarding cortisol levels in previous studies.

There are limitations to this study. First, there was no control group. However, considering the purpose of the study, the absence of a control group did not decrease this value. Second, because of the small volume and higher level of blood contamination in UWS samples compared with SWS (Kang et al., 2018), the number of UWS samples included in the final analyses was insufficient. However, this study's results suggested the possibility of using SWS in patients with BMS who may present low salivary flow. Third, the transferrin level in saliva samples was an indirect surrogate biomarker for measuring the degree of blood contamination. Further studies are needed to identify better biomarkers for detecting blood contamination in saliva samples and their thresholds for selecting appropriate saliva samples. Thus, additional salivary biomarker studies with considering blood contamination of saliva samples in a larger number of patients are required.

## 5 CONCLUSIONS

The results of the present study showed that each type BMS patients including the intermediate group had different clinical characteristics, which should be considered for more efficient evaluation and management. Additionally, cortisol and DHEA levels could be promising salivary biomarkers for differentiating between primary and secondary BMS.

## AUTHOR CONTRIBUTIONS

**Hong‐Seop Kho:** Conceptualization; data curation; formal analysis; funding acquisition; investigation; methodology; project administration; resources; supervision; validation; writing – original draft; writing – review and editing. **Mi‐Sun Kong:** Conceptualization; data curation; formal analysis; investigation; methodology; writing – original draft; writing – review and editing. **Moon‐Jong Kim:** Conceptualization; data curation; formal analysis; investigation; methodology; writing – original draft; writing – review and editing. **Yoon‐Young Kim:** Data curation; investigation; methodology; writing – review and editing. **Ji‐Youn Chang:** Methodology; data curation; investigation; writing – review and editing.

## FUNDING INFORMATION

This research was supported by the National Research Foundation of Korea (NRF) grant funded by the Korea government (MSIT) (No. 2019R1A2C1002437 and 2022R1A2C2003528).

## CONFLICT OF INTEREST STATEMENT

The authors declare no conflict of interest.

## INFORMED CONSENT

Informed consent was obtained from all individual participants included in the study.

## Supporting information


Tables S1–S7

## Data Availability

The data used and/or analyzed during the current study to support the findings are available from the corresponding author on reasonable request.
