# Supplementary material for: Clinical characteristics and salivary biomarkers of burning mouth syndrome
Source: Oral Dis. 2024 Apr 15;30(8):5360–9. doi: 10.1111/odi.14959 (PMC11610668; doi:10.1111/odi.14959)
Supplement: Supplementary file 1 — Tables S1–S7 [file ODI-30-5360-s001.docx]

**Supplementary materials**

**Supplementary tables**

TABLE S1 Summary of local and systemic factors in patients with intermediate and secondary BMS

| Local and systemic factors | Intermediate group (n) | Secondary group (n) |
| --- | --- | --- |
| Local |  |  |
| Symptoms remained after taking medication | 13^a^ |  |
| Symptoms relieved after taking medication |  | 5^b^ |
| Systemic |  |  |
| Under medical care | 12 |  |
| Not under medical care |  | 8^c^ |
| Local and systemic combined |  |  |
| Under medical care | 2^d^ |  |
| Not under medical care |  | 8 |
| Total | 27 | 21 |

BMS, burning mouth syndrome

^a^ Patients with no improvement in oral symptoms following the use of antifungal agents and/or topical steroid agents

^b^ Patients with improvement in oral symptoms following the use of antifungal agents

^c^ Patients with abnormal lab. findings

^d^ Patients with no improvement in oral symptoms following the use of antifungal agents and/or topical steroid agents and under medical care

TABLE S2 BMI and level of education of patients with burning mouth symptoms; *n* (%)

|  | Primary  (*n* = 50) | Intermediate  (*n* = 27) | Secondary  (*n* = 21) | *P* value | *P* value | | |
| --- | --- | --- | --- | --- | --- | --- | --- |
|  |  |  |  |  | P vs S | P vs I | S vs I |
| BMI (kg/m^2^) | (n = 49)^a^ | (n = 27) | (n = 19)^a^ |  |  |  |  |
| Normal (< 25) | 41 (83.7) | 20 (74.1) | 17 (82.1) | 0.621 | 1 | 0.445 | 0.501 |
| Overweight (≥ 25, < 30) | 7 (14.3) | 5 (18.5) | 2 (14.7) |  |  |  |  |
| Obesity (≥ 30) | 1 (2.0) | 2 (7.4) | 0 (3.2) |  |  |  |  |
| Level of education (years) | (n = 50) | (n = 25)^b^ | (n = 20)^b^ |  |  |  |  |
| 0 - 6 | 8 (16.0) | 4 (16.0) | 7 (35.0) | 0.203 | 0.197 | 0.328 | 0.245 |
| 7 – 9 | 7 (14.0) | 8 (32.0) | 3 (15.0) |  |  |  |  |
| 10 - 12 | 20 (40.0) | 7 (28.0) | 8 (40.0) |  |  |  |  |
| > 12 | 15 (30.0) | 6 (24.0) | 2 (10.0) |  |  |  |  |

BMI, body mass index; I, intermediate type of burning mouth syndrome (BMS); P, primary type of BMS; S, secondary type of BMS

^a^ One patient in the primary group and two in the secondary group did not answer the question about their height.

^b^ Two patients in the intermediate group and one in the secondary group did not answer the question about their level of education.

Pearson’s chi-square or Fisher’s exact tests were used to analyze differences.

TABLE S3-1 Self-reported initiating factors in patients with burning mouth symptoms; *n* (%)

| Initiating factors | Primary  (*n* = 50) | Intermediate  (*n* = 27) | Secondary  (*n* = 21) | *P* value | *P* value | | |
| --- | --- | --- | --- | --- | --- | --- | --- |
|  |  |  |  |  | P vs S | P vs I | S vs I |
| Trauma in the orofacial region | 0 (0) | 0 (0) | 0 (0) | - | - | - | - |
| Surgery | 0 (0) | 2 (7.4) | 1 (4.8) | 0.114 | 0.296 | 0.120 | 1 |
| Cold and/or flu | 5 (10.0) | 1 (3.7) | 2 (9.5) | 0.626 | 1 | 0.417 | 0.574 |
| Dental procedure | 7 (14.0) | 8 (29.6) | 2 (9.5) | 0.143 | 0.716 | 0.098 | 0.152 |
| Denture wearing | 1 (2.0) | 0 (0) | 1 (4.8) | 0.458 | 0.507 | 1 | 0.438 |
| Initiation of medication | 3 (6.0) | 3 (11.1) | 0 (0) | 0.399 | 0.550 | 0.659 | 0.246 |
| Food intake | 6 (12.0) | 0 (0) | 3 (14.3) | 0.117 | 1 | 0.085 | 0.077 |
| Stressful life event | 13 (26.0) | 7 (25.9) | 2 (9.5) | 0.277 | 0.202 | 0.994 | 0.264 |
| Systemic disease | 8 (16.0) | 3 (11.1) | 8 (38.1) | 0.044* | 0.062 | 0.738 | 0.040* |
| Unknown | 17 (34.0) | 10 (37.0) | 7 (33.3) | 0.954 | 0.957 | 0.790 | 0.790 |
| Etc. | 10 (20.0) | 5 (18.5) | 5 (23.8) | 0.899 | 0.756 | 0.876 | 0.792 |

I, intermediate type of burning mouth syndrome (BMS); P, primary type of BMS; S, secondary type of BMS

Pearson’s chi-square or Fisher’s exact tests were used to analyze differences.

* *P* < 0.05

TABLE S3-2 Self-reported aggravating factors in patients with burning mouth symptoms; *n* (%)

| Aggravating factors | Primary  (*n* = 50) | Intermediate  (*n* = 27) | Secondary  (*n* = 21) | *P* value | *P* value | | |
| --- | --- | --- | --- | --- | --- | --- | --- |
|  |  |  |  |  | P vs S | P vs I | S vs I |
| Spicy food | 36 (72.0) | 17 (62.9) | 13 (61.9) | 0.603 | 0.401 | 0.414 | 0.940 |
| Hot food | 30 (60.0) | 13 (48.2) | 9 (42.9) | 0.349 | 0.185 | 0.318 | 0.715 |
| Salty food | 18 (36.0) | 3 (11.1) | 7 (33.3) | 0.060 | 0.830 | 0.019* | 0.081 |
| Sweet food | 1 (2.0) | 0 (0) | 1 (4.8) | 0.458 | 0.507 | 1 | 0.438 |
| Toothpaste | 16 (32.0) | 5 (18.5) | 2 (9.5) | 0.097 | 0.047* | 0.205 | 0.445 |
| Gargling agent | 9 (18.0) | 5 (18.5) | 1 (4.8) | 0.347 | 0.262 | 1 | 0.211 |
| Tension or fatigue | 20 (40.0) | 13 (48.2) | 12 (57.1) | 0.402 | 0.185 | 0.491 | 0.536 |
| Feeling of anger | 6 (12.0) | 10 (37.0) | 1 (4.8) | 0.009* | 0.665 | 0.010* | 0.013* |
| Smoking | 0 (0) | 0 (0) | 0 (0) | - | - | - | - |
| Drinking alcohol | 4 (8.0) | 0 (0) | 0 (0) | 0.177 | 0.312 | 0.291 | - |
| Drinking soda | 5 (10.0) | 0 (0) | 1 (4.8) | 0.244 | 0.662 | 0.156 | 0.438 |
| Etc. | 9 (18.0) | 6 (22.2) | 5 (23.8) | 0.826 | 0.745 | 0.655 | 1 |

I, intermediate type of burning mouth syndrome (BMS); P, primary type of BMS; S, secondary type of BMS

Pearson’s chi-square or Fisher’s exact tests were used to analyze differences.

* *P* < 0.05

TABLE S3-3 Self-reported relieving factors in patients with burning mouth symptoms; *n* (%)

| Relieving factors | Primary  (*n* = 50) | Intermediate  (*n* = 27) | Secondary  (*n* = 21) | *P* value | *P* value | | |
| --- | --- | --- | --- | --- | --- | --- | --- |
|  |  |  |  |  | P vs S | P vs I | S vs I |
| Cold food | 20 (40.0) | 10 (37.0) | 3 (14.3) | 0.102 | 0.035* | 0.799 | 0.078 |
| Hot food | 7 (14.0) | 2 (7.4) | 3 (14.3) | 0.715 | 1 | 0.481 | 0.641 |
| Sweet food | 5 (10.0) | 3 (11.1) | 4 (19.1) | 0.609 | 0.435 | 1 | 0.638 |
| Toothpaste | 6 (12.0) | 0 (0) | 1 (4.8) | 0.135 | 0.665 | 0.085 | 0.438 |
| Gargling agent | 4 (8.0) | 3 (11.1) | 3 (14.3) | 0.683 | 0.415 | 0.691 | 1 |
| While concentrating | 8 (16.0) | 3 (11.1) | 4 (19.1) | 0.762 | 0.740 | 0.738 | 0.683 |
| Chewing gum | 12 (24.0) | 9 (33.3) | 5 (23.8) | 0.642 | 0.986 | 0.380 | 0.471 |
| Etc. | 9 (18.0) | 6 (22.2) | 3 (14.3) | 0.835 | 1 | 0.655 | 0.712 |

I, intermediate type of burning mouth syndrome (BMS); P, primary type of BMS; S, secondary type of BMS

Pearson’s chi-square or Fisher’s exact tests were used to analyze differences.

* *P* < 0.05

TABLE S4 Psychological analysis of patients with burning mouth symptoms; Median [IQR]

| SCL-90-R (t-score) | Primary  (*n* = 50) | Intermediate  (*n* = 26)^a^ | Secondary  (*n* = 21) | *P* value | *P* value | | |
| --- | --- | --- | --- | --- | --- | --- | --- |
|  |  |  |  |  | P vs S | P vs I | S vs I |
| Somatization | 43.0 [7.0] | 48.0 [13.8] | 45.0 [9.5] | 0.208 | 0.507 | 0.078 | 0.379 |
| Obsessive-compulsive | 42.0 [9.0] | 45.0 [14.5] | 40.0 [13.5] | 0.660 | 0.975 | 0.345 | 0.584 |
| Interpersonal sensitivity | 40.0 [8.0] | 43.0 [9.0] | 39.0 [8.5] | 0.463 | 0.694 | 0.223 | 0.464 |
| Depression | 43.0 [9.5] | 44.0 [13.3] | 45.0 [12.5] | 0.696 | 0.605 | 0.417 | 0.881 |
| Anxiety | 43.0 [8.0] | 43.0 [10.0] | 41.0 [9.0] | 0.921 | 0.944 | 0.708 | 0.747 |
| Hostility | 40.0 [7.0] | 43.0 [12.0] | 43.0 [9.0] | 0.234 | 0.175 | 0.159 | 0.948 |
| Phobic anxiety | 42.0 [8.0] | 45.0 [10.8] | 42.0 [3.5] | 0.194 | 0.698 | 0.107 | 0.133 |
| Paranoid ideation | 40.0 [4.0] | 40.0 [4.8] | 40.0 [4.0] | 0.801 | 0.529 | 0.695 | 0.803 |
| Psychoticism | 43.0 [7.0] | 43.0 [9.5] | 41.0 [11.0] | 0.962 | 0.854 | 0.908 | 0.771 |
| Global severity index | 41.5 [7.0] | 45.0 [10.3] | 43.0 [11.0] | 0.454 | 0.618 | 0.205 | 0.599 |
| Positive symptom distress index | 41.0 [6.0] | 45.0 [11.5] | 43.0 [10.0] | 0.008* | 0.105 | 0.002* | 0.497 |
| Positive symptom total | 43.0 [12.5] | 42.5 [19.3] | 43.0 [12.0] | 0.901 | 0.650 | 0.891 | 0.748 |

I, intermediate type of burning mouth syndrome (BMS); IQR, interquartile range; P, primary type of BMS; S, secondary type of BMS; SCL-90-R, Symptom Checklist 90-Revised

^a^ One patient in the secondary group did not answer the SCL-90-R test.

The Kruskal-Wallis test was used for continuous variables to analyze the differences across the three groups, and the Mann-Whitney U test was used for continuous variables between the groups.

* *P* < 0.05

TABLE S5 Questionnaire for sleep disturbance in patients with burning mouth symptoms; Median [IQR], *n* (%)

|  | Primary  (*n* = 50) | Intermediate  (*n* = 27) | Secondary  (*n* = 21) | *P* value | *P* value | | |
| --- | --- | --- | --- | --- | --- | --- | --- |
|  |  |  |  |  | P vs S | P vs I | S vs I |
| Quality of sleep (VAS) | 6.0 [3.5]^a^ | 5.0 [2.0] | 5.0 [4.3] | 0.251 | 0.162 | 0.640 | 0.122 |
| ISI |  |  |  |  |  |  |  |
| Initial | 2.0 [1.0] | 2.0 [1.0] | 2.0 [3.0] | 0.560 | 0.323 | 0.503 | 0.652 |
| Middle | 3.0 [1.0] | 3.0 [1.0] | 3.0 [2.0] | 0.751 | 0.922 | 0.479 | 0.553 |
| Terminal | 3.0 [2.0] | 3.0 [1.0] | 3.0 [2.0] | 0.858 | 0.591 | 0.745 | 0.872 |
| Satisfaction | 3.0 [2.0] | 4.0 [1.0] | 3.0 [2.5] | 0.834 | 0.572 | 0.991 | 0.607 |
| Interference | 2.0 [1.0] | 2.0 [1.0] | 2.0 [1.5] | 0.985 | 0.921 | 0.868 | 0.966 |
| Noticeability | 2.0 [2.0] | 2.0 [2.0] | 2.0 [2.5] | 0.903 | 0.720 | 0.712 | 0.922 |
| Distress | 2.0 [3.0] | 2.0 [1.0] | 2.0 [2.0] | 0.609 | 0.773 | 0.328 | 0.546 |
| Total | 9.0 [9.0] | 11.0 [7.0] | 8.0 [14.5] | 0.718 | 0.733 | 0.470 | 0.539 |
| No insomnia (0-7) | 15 (30.0) | 4 (14.8) | 9 (42.9) | 0.256 | 0.385 | 0.500 | 0.059 |
| Subthreshold insomnia (8-14) | 22 (44.0) | 16 (59.3) | 5 (23.8) |  |  |  |  |
| Clinical moderate (15-21) | 7 (14.0) | 4 (15.8) | 3 (14.3) |  |  |  |  |
| Clinical severe (22-28) | 6 (12.0) | 3 (11.1) | 4 (19.1) |  |  |  |  |

I, intermediate type of burning mouth syndrome (BMS); IQR, interquartile range; ISI, insomnia severity index; P, primary type of BMS; S, secondary type of BMS; VAS, visual analog scale

Quality of sleep was analyzed using VAS (0-10, with 10 being cannot sleep at all and 0 being no sleep loss).

The ISI questionnaire was used to evaluate the severity of insomnia.

^a^ One patient in the primary group did not answer about the quality of sleep.

The Kruskal-Wallis test was used for continuous variables to analyze the differences across the three groups, and the Mann-Whitney U test was used for continuous variables between the groups. Fisher’s exact test was used to analyze differences in categorical variables.

TABLE S6-1 Correlations between the intensities of oral symptoms and results of salivary biomarker analyses on unstimulated whole saliva samples; correlation coefficients (*r_s_*)^a^

| (*n* = 56) | Burning | Aching | Stinging | Numbness | Taste disturbance | Xerostomia | Sore throat | Eff-Life |
| --- | --- | --- | --- | --- | --- | --- | --- | --- |
| Total protein | -0.195 | 0.022 | 0.015 | -0.087 | 0.037 | 0.072 | 0.114 | -0.080 |
| CRP | 0.100 | -0.215 | -0.068 | -0.270 | -0.211 | 0.084 | -0.163 | 0.002 |
| IL-1β | -0.017 | -0.104 | 0.061 | 0.119 | -0.213 | -0.151 | -0.204 | -0.093 |
| IL-6 | 0.080 | -0.133 | -0.200 | 0.203 | 0.086 | -0.062 | 0.273 | 0.127 |
| 8-OHdG | -0.129 | -0.099 | 0.027 | 0.112 | 0.056 | -0.013 | 0.126 | -0.076 |
| MDA | 0.013 | -0.190 | -0.093 | -0.126 | -0.328 | -0.202 | -0.204 | -0.002 |
| TAC | -0.112 | -0.031 | 0.139 | 0.084 | -0.063 | -0.011 | 0.116 | 0.015 |
| Cortisol | 0.096 | -0.306 | -0.210 | -0.096 | -0.111 | 0.033 | -0.037 | 0.106 |
| DHEA | 0.066 | 0.019 | 0.072 | -0.107 | -0.142 | -0.008 | -0.080 | 0.247 |
| C/D ratio | -0.036 | -0.212 | -0.135 | 0.076 | 0.135 | 0.022 | -0.002 | -0.197 |
| Progesterone | 0.017 | 0.034 | -0.051 | -0.132 | 0.051 | 0.116 | 0.113 | -0.007 |
| 17β-Estradiol | -0.036 | -0.194 | -0.042 | -0.088 | -0.017 | 0.122 | -0.032 | -0.024 |

CRP, C-reactive protein; DHEA, dehydroepiandrosterone; IL, interleukin; MDA, malondialdehyde; 8-OHdG, 8-hydroxy-2’-deoxyguanosine; C/D ratio, cortisol/DHEA ratio

^a^ Correlations were analyzed using Spearman’s correlation test with Bonferroni correction.

Bonferroni corrected level (*P* < 0.000521) was used to evaluate significance.

TABLE S6-2 Correlations between the intensities of oral symptoms and results of salivary biomarker analyses on stimulated whole saliva samples; correlation coefficients (*r_s_*)^a^

| (*n* = 92) | Burning | Aching | Stinging | Numbness | Taste disturbance | Xerostomia | Sore throat | Eff-Life |
| --- | --- | --- | --- | --- | --- | --- | --- | --- |
| Total protein | 0.030 | 0.096 | -0.035 | -0.044 | -0.036 | 0.058 | 0.054 | 0.096 |
| CRP | 0.024 | -0.132 | -0.014 | -0.138 | -0.046 | -0.055 | -0.074 | -0.069 |
| IL-1β | -0.004 | 0.036 | -0.068 | 0.104 | -0.047 | -0.004 | -0.137 | 0.002 |
| IL-6 | 0.112 | -0.094 | 0.050 | 0.010 | -0.066 | -0.180 | 0.107 | 0.016 |
| 8-OHdG | -0.069 | 0.112 | -0.042 | 0.020 | 0.055 | 0.063 | -0.008 | -0.051 |
| MDA | -0.004 | -0.192 | -0.089 | -0.095 | -0.234 | -0.106 | -0.168 | -0.070 |
| TAC | -0.037 | 0.027 | 0.034 | 0.073 | -0.002 | 0.047 | -0.004 | 0.033 |
| Cortisol | 0.020 | -0.426^*^ | -0.056 | -0.098 | -0.096 | -0.086 | -0.115 | -0.031 |
| DHEA | -0.053 | -0.075 | 0.029 | -0.015 | -0.158 | -0.119 | -0.218 | -0.051 |
| C/D ratio | 0.057 | -0.180 | -0.091 | -0.022 | 0.049 | 0.054 | 0.126 | -0.016 |
| Progesterone | -0.102 | -0.117 | -0.102 | -0.134 | -0.112 | 0.029 | -0.172 | -0.207 |
| 17β-Estradiol | -0.055 | -0.021 | -0.079 | -0.054 | -0.057 | -0.001 | -0.102 | -0.051 |

CRP, C-reactive protein; DHEA, dehydroepiandrosterone; IL, interleukin; MDA, malondialdehyde; 8-OHdG, 8-hydroxy-2’-deoxyguanosine; C/D ratio, cortisol/DHEA ratio

^a^ Correlations were analyzed by Spearman correlation test with Bonferroni correction.

Bonferroni corrected level (*P* < 0.000521) was used to evaluate significance. * *P* < 0.000521

TABLE S7-1 Correlations between the results of the Symptom Checklist-90-Revised (SCL-90-R) and those of salivary biomarker analyses on unstimulated whole saliva samples; correlation coefficients (*r_s_*)^a^

| (*n* = 56) | SOM | O-C | IS | DEP | ANX | HOS | PHOB | PAR | PSY | GSI | PSDI | PST |
| --- | --- | --- | --- | --- | --- | --- | --- | --- | --- | --- | --- | --- |
| Total protein | -0.024 | -0.185 | -0.132 | -0.118 | -0.136 | -0.016 | -0.207 | 0.152 | -0.001 | -0.120 | -0.169 | -0.103 |
| CRP | 0.151 | 0.153 | 0.103 | 0.125 | 0.199 | 0.060 | 0.161 | 0.049 | 0.156 | 0.178 | 0.127 | 0.130 |
| IL-1β | -0.176 | -0.241 | -0.255 | -0.198 | -0.151 | -0.162 | -0.192 | -0.080 | -0.284 | -0.219 | -0.253 | -0.170 |
| IL-6 | 0.078 | -0.018 | -0.063 | -0.006 | 0.031 | -0.046 | -0.216 | -0.032 | -0.113 | -0.038 | 0.000 | -0.088 |
| 8-OHdG | 0.029 | 0.025 | 0.028 | 0.138 | 0.137 | -0.039 | 0.063 | 0.035 | 0.052 | 0.042 | -0.048 | 0.079 |
| MDA | -0.058 | -0.017 | -0.040 | 0.140 | 0.144 | 0.093 | 0.187 | 0.091 | -0.039 | 0.068 | -0.084 | 0.108 |
| TAC | -0.031 | -0.095 | -0.150 | 0.084 | -0.161 | -0.056 | -0.008 | -0.115 | -0.082 | -0.085 | -0.141 | -0.076 |
| Cortisol | -0.199 | 0.032 | 0.044 | -0.092 | 0.145 | -0.002 | -0.034 | 0.068 | -0.007 | 0.011 | 0.025 | -0.019 |
| DHEA | -0.160 | -0.100 | -0.094 | 0.021 | 0.042 | -0.020 | 0.090 | -0.027 | -0.265 | -0.063 | 0.021 | -0.096 |
| C/D ratio | 0.023 | 0.136 | 0.118 | -0.077 | 0.007 | 0.046 | -0.133 | 0.130 | 0.259 | 0.070 | -0.008 | 0.091 |
| Progesterone | 0.073 | -0.042 | -0.093 | 0.074 | 0.223 | -0.051 | 0.170 | -0.070 | 0.115 | 0.040 | 0.014 | 0.094 |
| 17β-Estradiol | 0.123 | 0.131 | 0.157 | 0.132 | 0.217 | 0.121 | 0.147 | 0.042 | 0.148 | 0.141 | 0.146 | 0.159 |

SOM, somatization; O-C, obsessive-compulsive; IS, interpersonal sensitivity; DEP, depression; ANX, anxiety; HOS, hostility; PHOB, phobic anxiety; PAR, paranoid ideation; PSY, psychoticism; GSI, global severity index; PSDI, positive symptom distress index; PST, positive symptom total; CRP, C-reactive protein; DHEA, dehydroepiandrosterone; IL, interleukin; MDA, malondialdehyde; 8-OHdG, 8-hydroxy-2’-deoxyguanosine; C/D ratio, cortisol/DHEA ratio

^a^ Correlations were analyzed using Spearman’s correlation test with Bonferroni correction.

Bonferroni corrected level (*P* < 0.000347) was used to evaluate significance.

TABLE S7-2 Correlations between the results of the Symptom Checklist-90-Revised (SCL-90-R) and those of salivary biomarker analyses on stimulated whole saliva samples; correlation coefficients (*r_s_*)^a^

| (*n* = 92) | SOM | O-C | IS | DEP | ANX | HOS | PHOB | PAR | PSY | GSI | PSDI | PST |
| --- | --- | --- | --- | --- | --- | --- | --- | --- | --- | --- | --- | --- |
| Total protein | 0.016 | -0.171 | -0.141 | 0.010 | -0.065 | 0.009 | 0.056 | -0.078 | -0.184 | -0.067 | 0.084 | -0.120 |
| CRP | 0.125 | 0.079 | -0.009 | 0.119 | 0.148 | 0.007 | 0.081 | 0.075 | 0.056 | 0.135 | 0.160 | 0.042 |
| IL-1β | -0.067 | -0.085 | -0.084 | 0.011 | -0.047 | -0.104 | -0.115 | 0.035 | -0.074 | -0.020 | 0.077 | -0.104 |
| IL-6 | -0.065 | -0.169 | -0.178 | -0.054 | -0.006 | -0.163 | -0.035 | -0.085 | -0.114 | -0.106 | -0.081 | -0.146 |
| 8-OHdG | 0.056 | -0.064 | 0.028 | -0.001 | 0.077 | -0.078 | -0.014 | 0.069 | 0.029 | 0.013 | 0.095 | -0.034 |
| MDA | -0.129 | -0.088 | -0.103 | 0.039 | 0.053 | -0.087 | 0.037 | -0.053 | -0.052 | -0.039 | -0.004 | -0.043 |
| TAC | -0.046 | -0.144 | -0.009 | -0.057 | -0.198 | -0.002 | -0.187 | -0.039 | -0.182 | -0.105 | -0.029 | -0.163 |
| Cortisol | -0.229 | -0.028 | 0.028 | -0.108 | 0.045 | -0.065 | -0.085 | 0.059 | 0.046 | -0.046 | -0.131 | -0.017 |
| DHEA | -0.240 | -0.240 | -0.142 | -0.116 | -0.054 | -0.230 | -0.062 | -0.101 | -0.195 | -0.191 | -0.019 | -0.223 |
| C/D ratio | 0.030 | 0.238 | 0.199 | 0.056 | 0.074 | 0.160 | 0.000 | 0.176 | 0.235 | 0.150 | -0.056 | 0.193 |
| Progesterone | -0.031 | -0.111 | -0.053 | -0.050 | -0.007 | -0.181 | -0.037 | -0.101 | -0.038 | -0.072 | -0.023 | -0.105 |
| 17β-Estradiol | 0.077 | 0.082 | 0.131 | 0.150 | 0.111 | -0.005 | 0.178 | 0.121 | 0.030 | 0.120 | 0.201 | 0.056 |

SOM, somatization; O-C, obsessive-compulsive; IS, interpersonal sensitivity; DEP, depression; ANX, anxiety; HOS, hostility; PHOB, phobic anxiety; PAR, paranoid ideation; PSY, psychoticism; GSI, global severity index; PSDI, positive symptom distress index; PST, positive symptom total; CRP, C-reactive protein; DHEA, dehydroepiandrosterone; IL, interleukin; MDA, malondialdehyde; 8-OHdG, 8-hydroxy-2’-deoxyguanosine; C/D ratio, cortisol/DHEA ratio

^a^ Correlations were analyzed using Spearman’s correlation test with Bonferroni correction

Bonferroni corrected level (*P* < 0.000347) was used to evaluate significance.
